# Food Application of Orange Seed Powder through Incorporation in Wheat Flour to Boost Vitamin and Mineral Profiles of Formulated Biscuits

**DOI:** 10.1155/2023/6654250

**Published:** 2023-11-16

**Authors:** Ashiq Hussain, Salah Laaraj, Tusneem Kausar, Aziz Tikent, Hanane Azzouzi, Samina Kauser, Qurat Ul An, Aqsa Iqbal, Saima Akram, Rizwan Nisar, Ayesha Najam, Haya Fatima, Shazia Yaqub, Kaoutar Elfazazi

**Affiliations:** ^1^Institute of Food Science and Nutrition, University of Sargodha, Sargodha, Pakistan; ^2^Agri-Food Technology and Quality Laboratory, Regional Center of Agricultural Research of Tadla, National Institute of Agricultural Research (INRA), Avenue Ennasr, BP 415 Rabat Principal, 10090 Rabat, Morocco; ^3^Laboratoire d'Amélioration des Productions agricoles, Biotechnologie & Environnement (LAPABE), Faculté des Sciences, Université Mohammed Premier, BP: 717, 60000 Oujda, Morocco; ^4^Punjab Food Authority, Lahore, Punjab, Pakistan; ^5^PMAS Arid Agriculture University, Rawalpindi, Punjab, Pakistan

## Abstract

The significance of conducting research for its application has been noted as a result of the rising global food production and waste generation. As a result, there is increasing interest in fruits and vegetable seeds that contain bioactive chemicals, such as those that are obtained from orange seeds. In the current work, orange seed powder replaced wheat flour at 0, 2.5, 5, 7.5, and 10% levels, to observe changes in physicochemical features of developed biscuits. Proximate analysis of orange seed powder and wheat flour revealed that orange seed powder has high fat, fiber, protein, and ash contents as compared to wheat flour, whereas moisture contents in wheat flour were high. In developed biscuits, the highest values (percentage) of ash (9.68 ± 0.04), fiber (6.79 ± 0.12), protein (10.42 ± 0.25), and fat (36.90 ± 0.55) were found in biscuits developed with 10% orange seed powder. Orange seed powder was a comparatively good source of both macro and micro minerals, as compared to wheat flour. High contents of selenium (5.32 ± 0.03), iron (2.12 ± 0.05), zinc (3.88 ± 0.12), and manganese (2.25 ± 0.04) mg/100 g, present in orange seed powder, were the prominent findings of this research work, as wheat flours were observed to be deficient in these trace minerals. Contents of calcium, magnesium, potassium, zinc, manganese, zinc, and selenium in control biscuits were found 20.51 ± 0.08, 17.29 ± 0.04, 46.12 ± 0.05, 1.06 ± 0.01, 1.97 ± 0.01, 0.12 ± 0.01, and 0.11 ± 0.01 mg/100 g, respectively, and replacement of wheat flour with 10% orange seed powder increased values of these minerals to 103.90 ± 0.35, 44.35 ± 0.50, 71.29 ± 0.32, 2.59 ± 0.4, 2.75 ± 0.02, 1.31 ± 0.01, and 2.02 ± 0.05 mg/100 g, respectively. Vitamins E and K, which were not detected in wheat flour, were present in orange powder in high amount, whereas B group vitamins, which were also present in wheat flour, were observed in significantly high quantities in orange seed powder. Increment in vitamin A, D, E, K, and B complexes was significant as a result of orange seed powder supplementation, except for vitamins B1 and B2, which were slightly decreased. Sensory evaluation revealed that a 5% replacement of orange seed powder provided good quality biscuits with acceptable colour, flavor, taste, texture, and overall acceptability. Orange seed powder could prove an important ingredient in the baking industry with the potential of promoting the nutritional value of foods.

## 1. Introduction

As the world emerges from the destructive pandemic period, eating the right foods can help to develop and strengthen adaptive immunity. Plant-based foods, due to the presence of functional and nutraceutical components, must be part of one's daily diet. In the current post-COVID-19 situation, a sufficient supply of healthy foods that are balanced with pharmaceutical foods may play a fundamental role in boosting the immune systems of the populaces [1]. In accordance with this functional food covenant, the world is looking for fresh, nutritious food items that are rich in bioactive ingredients like fiber, minerals, vital amino acids, and phenols [2]. Worldwide consumption of bakery products like biscuits and cookies is high; however, these foods lack adequate nutritional value, due to the use of refined white flour [3]. To enhance these food products with phytochemicals like phenolics and flavonoids, vitamins and minerals, carotenoids and natural colourants, and fiber, powdered plants, herbs, fruits, and vegetables may be used as functional ingredients [4]. Different parts of plants may be extracted to obtain condensed phytochemicals, which exhibit strong antioxidant potential upon consumption in different forms [5, 6].

The most widely planted fruit tree in the world is the sweet orange (*Citrus sinensis*). Orange seeds are typically found lodged in the middle of the fruit, close to the juice sacs. The flattened, angular seeds are green to pale white in colour [7]. Orange by-products, including the seeds, are extremely concerning trash that can seriously pollute the environment. However, due to their nutritive and technological qualities, orange fruit wastes are a potential material in the food sectors [8, 9]. Orange seed flour's great capacity to absorb water makes it a good candidate for usage as a functional ingredient in baked products [10]. One by-product of fruit consumption and industrial processing is still the seeds. The perceived value of orange seeds as a commodity is low. As a result, the seeds are frequently thrown away, causing environmental problems such as the production of greenhouse gases as well as financial losses for the food business [11]. Oranges come in many different types, including common oranges, navel oranges, blood oranges, and acidless oranges. Each variety is recognized by the others by its distinctive flavor, appearance, texture, and ability to produce juice [12, 13]. More than 140 countries are said to grow citrus fruit, and in recent years, both production and commerce have steadily increased. Approximately one-third of all citrus fruit produced worldwide is utilized for processing. The global production of citrus family fruits was estimated to reach 158.49 million tonnes worldwide in 2020 on an area of 1.07 hectares [14].

Citrus fruits have been categorized as fruits of worldwide economic importance because of their ability to promote health by providing essential nutrients. Citrus fruits are known for being salt, cholesterol, and sugar-free [15]. According to studies, citrus fruits' high concentrations of dietary fiber, vitamin C, niacin, folic acid, thiamine, calcium, phosphorus, potassium, magnesium, and copper reduce the risk of developing cancer, heart disease, respiratory conditions, diet-related illnesses, and novel COVID-19 [16, 17]. In a recent analysis of studies that have been published on the bioactive and nutritional qualities of orange seeds, it was discovered that fatty acids, tocopherols, phytosterols, minerals, vitamins, and fiber were the key nutrients [18]. High contents of oil are present in orange seeds, and the oil is a good source of essential and unsaturated fatty acids like oleic and linoleic acids. The seeds also contain beneficial substances such as carotenoids and flavonoids, as antioxidants that inhibit the actions of free radicals, and these substances may have positive impacts on health [17].

Food by-product valorization has received a lot of attention recently. In the manufacturing process of juice, citrus fruits generate significant amounts of nonedible waste, up to 80%. Peel, pulp, and seeds are regarded as agricultural trash [18]. For the fruit processing business, using this waste is essential not only for financial reasons but also to lessen the potentially disastrous environmental effects that it could have [19]. To increase the sustainability of the food chain, the valorization of food by-products and wastes has emerged as a key study area. Utilizing resources in a circular manner entails creating gentle methods that convert food by-products into goods with added value without the use of organic solvents or prolonged washing and drying procedures [13, 18]. The current state of affairs can be linked to a lack of knowledge regarding the advantages of the seed. In order to maximize environmental protection, add value to those wastes, and offer a fantastic nutritious alternative. Due to their current extensive potential for usage in the food, pharmaceutical, and cosmetic industries, their utilization has become absolutely necessary [11].

In baked products, refined ingredients are frequently employed, resulting in excessive quantities of saturated fatty acids and sugars as well as a deficiency of vital nutrients such as minerals, vitamins, and dietary fibers [20]. Worldwide production and consumption of bakery foods developed from refined ingredients is very high due to their ready-to-eat nature and ease in development; however, recently, consumers have grown their interest towards formulated food items that found their source of phytochemicals from fruits and vegetables. Food ingredients that are powdered are easier to transport, store, process, incorporate, and use [4, 18]. Sweet orange seeds are excellent candidates for food fortification agents because of their high lipid, fiber, carotenoid, polyphenols, and protein contents. It may be a good idea to process, extract, refine, and use specific composites in fruit seeds that have high nutritional and biologically active components, in order to make use of and add value to the fruit seeds [21] To take advantage of the dietary and functional benefits of fiber, a broad variety of high nutritional fiber-formulated foods have been developed and sold for health-conscious consumers [22]. Wheat flour biscuits with lemon and orange by-product powder [23], biscuits by incorporation of mandarin peel powder [24], crackers from orange seeds [25], biscuits from orange peel powder [26], biscuits from citrus by-products [27], and biscuits through the incorporation of lemon pomace powder [28] have been reported earlier by recent studies. However, there were certain gaps, which were needed to be covered by exploring further the chemical composition of orange seed flour and comparing it with wheat flour. After careful evaluation of previous studies, it was noticed that no or few experiments were performed to evaluate essential minerals and vitamins present in orange seed powder and food products developed by the incorporation of orange seed powder.

Keeping in view these helpful and dominating facts about citrus wastes, the goal of the current research was to convert orange seeds into powder for the determination of vitamins and minerals, to create nutritional biscuits using different replacement levels of orange seeds powder, and to analyze the vitamins, minerals, proximate composition, and sensory parameters of these biscuits. The facts regarding the phytochemistry of orange seeds and the possible applications of orange seed powder in food products were taken into consideration when doing this, in order to produce good quality and acceptable bakery products with health-promoting potential.

## 2. Materials and Methods

### 2.1. Collection of Raw Material

Samples of orange fruit were collected from the Pakistan Citrus Research Institute in Sargodha, Pakistan. Based on size, the oranges were divided into three categories: small, medium, and large, and samples of uniform size without any damage were further taken for seed separation. For the creation of the biscuits, additional ingredients (straight-grade white flour, sugar, butter, and eggs) were bought from the Abu Junaid cash-and-carry superstore in Sargodha, Pakistan. Chemicals and reagents for analyses were provided by Sigma-Aldrich (Germany). To prevent any variance in outcomes, the identical trade reagents were used in each trial.

### 2.2. Orange Seed Powder Preparation

The orange fruits' seeds were removed by cutting them. The seeds were properly cleaned, put evenly on trays, and made ready for use by doing so. Orange seeds were cleaned in tap water. Orange seeds (20 kg) were soaked in tap water (1 : 10, seed: water) for 12 h. Thereafter, the hydrated seeds were boiled for 30 min. The samples were dehulled manually and winnowed. The trays having seeds were then dried in a hot air oven (Hitachi, HT-300 Japan) until the weight of the seeds remained consistent. Prior to the proximate, mineral, and vitamin analyses, the seeds were ground to a fine powder using a laboratory mill (42823CO, Hamburg) and kept in zip-top bags at room temperature.

### 2.3. Development of Formulated Biscuits with Various Replacement Levels of Orange Seed Powder

Wheat flour was replaced with orange seed powder at 0, 2.5, 5, 7.5, and 10% replacement levels for the development of different treatment biscuits. The method used to make the biscuits by Hussain et al. [29] was modified in a few key areas. To put it simply, ingredients were measured, combined, mixed well, and sheeted to create the batter. With the use of mould, biscuits were then moulded and set on stainless steel trays. A baking oven was used to bake the food for 20 minutes at 180°C. Wheat flour was used to make the biscuits, with varying amounts of orange seed powder substituted separately. For additional studies, prepared biscuits were placed on a lab shelf in polythene bags at room temperature.

### 2.4. Chemical Analysis of Orange Seed Powder and Developed Biscuits

#### 2.4.1. Proximate Analysis

According to the procedure prescribed by AACC [30], the proximate analysis of the wheat flour and orange seed powder was carried out for ash, fat, fiber, moisture, and protein contents, by following their respective methods. Similarly, the proximate analyses of orange seed powder-incorporated biscuits were determined by adopting the same procedures, with required modifications.

#### 2.4.2. Mineral Analysis

The approved AOAC [31] technique was used to determine the minerals. Sodium (Na) and potassium (K) concentrations were measured using the PFP 7 flame emission photometer. The standards utilized were NaCl and KCl. Atomic absorption spectrophotometer (S.P-300, Canada) measurements were made to assess the amounts of calcium (Ca), phosphorus (P), and iron (Fe). At wavelengths of 770 nm, 540 nm, and 520 nm, respectively, the absorbance of P, Ca, and Fe was measured. In mg/100 g, all values were expressed.

### 2.5. Determination of Vitamins

Using an atomic absorption spectrophotometer, the vitamins present in orange seed powder and developed biscuits were separated and detected in accordance with Ruales and Nair [32] and Nwozo and Nwawuba [33] approaches, with required modifications. Vitamin A, D, E, K, and B groups were examined in the sample. Unless otherwise specified, all of the compounds utilized for this analysis were of analytical grade.

### 2.6. Sensory Evaluation of Developed Biscuits

The created products that contained orange seed powder underwent sensory evaluation using the nine-point hedonic rating scale as described by Tsikritzi et al. [34]. A panel of 20 experts, with a combined average age of 45, were given sheets with scores ranging from 1 to 9, with 1 highly representing disapproval and 9 representing strong approval. The experts were given samples of biscuits with unique codes, and distilled water bottles were used to rinse and neutralize their mouths after each test. The collected, computed, and analyzed data were acquired.

### 2.7. Statistical Analyses

Results were reported as means ± standard deviations, and all analyses were carried out in triplicate to obtain triplicate determinations. The one-way ANOVA method was used for the statistical study. To distinguish between the mean numbers, Duncan's multiple-range test was utilized by following the protocols elaborated by Steel et al. [35].

## 3. Results and Discussion

### 3.1. Proximate Composition Analyses of Wheat Flour and Orange Seed Powder

Results presented in Table 1 compared the proximate composition of wheat flour and orange seed powder, and it was evident that orange seed powder has high fat, fiber, protein, and ash contents as compared to wheat flour, whereas moisture contents in wheat flour were high. Hussain et al. [29] performed the proximate analysis of white flour before the development of nutritional biscuits and provided values of moisture, ash, fat, fiber, and protein contents in white flour as 13.56, 1.05, 0.97, 0.72, and 10.11 mg/100 g, respectively. Comparing this proximate composition of wheat flour with that of orange seed powder, it was evident that orange seed powder was a good source of fat, fiber, protein, and ash contents.

Emojorho and Akubor [10] provided much similar findings for the proximate composition of orange seed powder, highlighting the high amount of ash, fat, fiber, and protein. Further, they compared the effect of different debittering methods on the proximate composition of orange seeds and reported that boiling for 40 minutes was an optimum tool for obtaining the best quality powder. In a related study by Uzama et al. [36], the results for the proximate composition of orange seeds showed that the seeds contain moisture (10.92%), ash (4.31%), crude fat (6.37%), crude fiber (1.67%), crude protein (2.36%), and carbohydrate (74.37%). This variation in the results might be due to the difference in orange varieties selected and pretreatments of the orange seeds, and both of these factors cause highly significant effect on the chemical composition of flours. In another study, Talens et al. [20] reported high contents of fiber and slightly lower contents of moisture, protein, and ash in the microwave and hot air oven-dried orange seed powder than reported in current findings.

Ojha and Thapa [24] developed healthy biscuits by mixing powdered dried mandarin peel with wheat flour. When mandarin peel powder and wheat flour were compared, it became clear that mandarin peel powder contained larger concentrations of fiber, ash, and fat, whereas wheat flour contained lower concentrations of moisture, protein, and sugar. Additionally, they stated that there are significant levels of polyphenols, carotenoids, and ascorbic acid in mandarin peel powder. In research by Jurasova and Kukurova [23], powders made from dried orange and lemon pomaces were used in chemical analyses, and the results corroborated the findings of the current study. They determined that the crude fiber content of the powdered lemon and orange pomace was 56.5% and 63.4%, respectively.

The seeds' potential for a longer shelf life is increased by the low moisture content of the seeds. The seeds' low moisture content provides compelling evidence that they can be processed (milled, mixed, and extracted for oil). By restricting moisture-dependent biochemical reactions and preventing the development of mould, the flour's low moisture content would boost its storage durability [12]. Orange seed types used in this study are promising sources for commercial vegetable oil extraction due to their high oil content. A sample's percentage of ash can be used to estimate its inorganic composition from which the mineral content can be deduced. High concentrations of different mineral elements are predicted to be present in samples with high proportions of ash content, which is predicted to haste up metabolic progressions and enhance growth and development [37]. As a result, adding either of the two kinds to any feed or food item for people or animals would help to reduce the lack of micronutrients in both plants and animals. The analytical approach employed for the experiment has a significant impact on the variation in protein levels in seeds. Orange seeds are one of the seeds with a high protein content when compared to other seeds because of their protein level. The seeds' high protein content makes them a useful source of commercial protein, which leads to their use in fertilizer, food, and the preparation of animal feed. The orange seeds had a very high fiber content; therefore, adding them to other low-energy items to increase the fiber content of such foods will be very important for meeting the requirement for fiber in food products [38]. Supportive findings were also reported by Yilmaz and Karaman [25], when they observed an increase in fiber contents of citrus seed-incorporated crackers.

### 3.2. Mineral Composition of Wheat Flour and Orange Seed Powder

Data presented in Table 2 provided important mineral amount present in wheat flour and orange seed powder, and it was observed that orange seed powder was a comparatively good source of both macro and micro minerals, as compared to wheat flour. High contents of selenium, manganese, iron, and zinc present in orange seed powder were the prominent findings of this research work, as wheat flours have been reported to be deficient in these trace minerals, and in different time periods, different experiments have been developed to meet these deficiencies not only in wheat flour but also in developed products. Findings of Suteu et al. [13] were useful in explaining the current outcomes regarding the mineral contents of orange seed powder, as that study presented similar findings. Results for the mineral composition of orange seeds reported in a related study by Uzama et al. [36] showed that the orange seeds contain Mn (0.13 mg/100 g), Cu (0.27 mg/100 g), Zn (0.63 mg/100 g), Ca (31.00 mg/100 g), Mg (1.02 mg/100 g), Na (55.56 mg/100 g), and K (57.50 mg/100 g), and these lower values of minerals than our findings might be due to the difference in cultivar, environment, soil conditions, pretreatments, and determining techniques.

Current study results were far higher than those El-Safy et al. [39] obtained when they looked at the nutritional profile of orange seed flours, as seed flour samples had sizable levels of P, Ca, Mg, K, Cu, Fe, and Zn, making them potentials for future dietary supplements, according to the results, which showed that the analyzed seed flours were thought to be major new mineral sources. However, the figures were less than the mineral level discovered by El-Adawy et al. [40], when they examined the mineral makeup of citrus seed flour and discovered that when combined with wheat flour, citrus seeds and flours were effective sources of the minerals K, Ca, P, Na, Fe, and Mg, with potential food applications in combination with wheat flour. Genetic alteration, soil type, climatic changes, soil nutrients, and other agricultural performs including fertilization, weed management, and land preparation are all contributing factors to the unexpectedly large range [41].

According to Umeta et al. [41], changes in soil nutrients, soil texture, land topography, fertilizer application, manure, genetic alteration, and kind of plant are what create variations in a plant's mineral composition and that of its bearings (fruits and seeds). The two seeds' high potassium concentration suggests that they may be used in food products for both humans and animals to help regulate pH, maintain cellular water balance, and process proteins and carbohydrates. Orange seeds are a great source of calcium, which is crucial for developing bones and teeth in children and pregnant women as well as for healthy blood circulation in adults. The role of zinc and iron has been vital for better immune functions [15].

The macronutrients that are crucial for human nutrition and that promote health are minerals. The findings of this investigation (Table 2) showed that Ca, Mg, K, Na, P, Mn, Fe, and Zn were present in orange seed powder. Manganese plays a crucial role in the delivery of oxygen to the cells, aids in cognitive function, and works as a cofactor for enzymes involved in the metabolism of fat, protein, and carbohydrates [33]. The body requires calcium, a key macronutrient, for many physiological and biochemical functions. Zinc is a well-known antioxidant and metallo-enzyme that is essential for the health of the immunological, reproductive, and central nervous systems. According to Hartley et al. [42], zinc deficiency in children is linked to dwarfism, respiratory infections, taste sensitivity, stunted growth, and increased diarrhea. Similar to how iron (Fe) is found in the haem of hemoglobin, magnesium (Mg) is located in the center of the porphyrin in the molecule responsible for photosynthesis. A vital metal in the human body, iron (Fe), is also the center of the haem group in hemoglobin, which gives blood its red colour [43]. The release of parathyroid hormones in critical organs and tissues, oxidative phosphorylation, typical muscular contraction, and relaxation and the transformation of vitamin D into its active form are all correlated with the presence of magnesium. Mg deficiency causes convulsions, while calcium is crucial for building the body's tissues and bones [44].

### 3.3. Vitamin Analysis of Wheat Flour and Orange Seed Powder

Vitamin analysis data of wheat flour and orange seed powder has been presented in Table 3, from where it was seen that vitamins E and K, which were not detected in wheat flour, were present in orange seed powder in a high amount. B group vitamins, which were also present in wheat flour, were observed in significantly high quantities in orange seed powder. From these results, it was evident that orange seed powder was a good source of both water-soluble and fat-soluble vitamins. According to Nwozo and Nwawuba [33], vitamins are a necessary component of food; deficiencies in vitamins A and D cause blindness and rickets in children, vitamin E has antioxidant properties, vitamin K is necessary to prevent blood loss in the event of injury deficiency, and vitamin K increases calcium deposition, which may cause coronary artery calcification and the onset of heart disease [42, 45].

Vitamin A deficiency causes dry skin and dermatitis-like effects. A lack of it causes development retardation, night blindness, or faulty perception in low light. With longer boiling times, the orange seed flours' vitamin D content exhibited varied behaviors. Vitamin D deficiency is linked to the nutritional illness' rickets and osteomalacia (softening of the bones). Vitamin E's main role is as an antioxidant, protecting other nutrients and preventing lipid peroxidation. Vitamin K has a role in blood coagulation [45–47].

Pyruvic acid must be converted to acetyl CoA in order to use thiamine. The vitamin is essential for promoting hunger, digestion, and growth. Thiamine contributes to the release of energy from the metabolism of carbohydrates. The metabolism of fat was affected. Thiamine deficiency results in beriberi, weight gain, loss of skin sensibility, edema in children (waterlogged skin), gastro intestinal problems, and cardiac failure [46, 47]. The signs and symptoms of riboflavin deficiency (also known as ariboflavinosis) include skin disorders, hyperemia (excess blood) and edema of the mouth and throat, angular stomatitis (lesions at the corners of the mouth), cheilosis (swollen, cracked lips), hair loss, reproductive issues, sore throat, itchy and red eyes, and liver and nervous system degeneration [48]. Glossitis, a disorder that results in enlarged tongues and lips as well as scaliness at the corners of the mouth, is brought on by riboflavin deficiency in men [46]. Niacin deficiency causes pellagra, which is also known as the 3D sickness due to its primary symptoms of diarrhea, dermatitis, and delirium [49]. Niacin treatment is necessary to prevent the development of dementia, dermatitis, diarrhea, and eventually mortality [50]. Vitamin B6 is used to prevent or treat vitamin B6 deficiency that is brought on by ailments like severe diarrhea, malabsorption, congenital metabolic dysfunction, hyperthyroidism, renal and hepatic disease, congestive heart failure, alcoholism, drug-induced conditions, and while pregnant or nursing [48]. Anaemia is a disorder brought on by folic acid deficiency. The creation of healthy red blood cells, which is crucial during periods of rapid growth during pregnancy and fetal development, depends on folate as well [51]. The creation and maintenance of healthy blood depend on folate. It works in conjunction with vitamin B12 to produce blood, preventing anaemia. The inability of erythrocytes to mature in the absence of vitamin B12 causes pernicious anaemia, which is characterized by weakness, exhaustion, glossitis, smoothness of the tongue, and sore and cracked lips [46].

Studies by Makpoul and Ibrahem [52] provided much similar results as the amount of these vitamins in wheat flour was quite lower than in quinoa flour and due to which wheat flour biscuits were deficient in these vitamins, which was overcome by replacing wheat flour with quinoa flour.

### 3.4. Proximate Composition of Orange Seed Powder-Incorporated Biscuits

Results for the approximate composition of biscuits containing orange seeds are shown in Table 4, and it is clear from these mean values that as the proportion of wheat flour used to replace the orange seed powder was increased, the ash, fat, fiber, and protein contents significantly increased while the moisture contents decreased. Highest values (percentage) of ash (9.68 ± 0.04), fiber (6.79 ± 0.12), protein (10.42 ± 0.25), and fat (36.90 ± 0.55) were found in biscuits developed with 10% orange seed powder.

In a food material, ash content is a nonorganic compound in the food that contains mineral content. Nutritionally, it helps in the process of metabolism of other organic compounds such as carbohydrates and fats [53]. In order to study the biscuits' chemical and functional qualities, Jurasova and Kukurova [23] produced biscuits with varying replacement levels of lemon and orange fiber compositions. According to the research, adding citrus waste part powders improved the rheological, technological, and dietary parameters of manufactured biscuits.

Ojha and Thapa [24] replaced wheat flour with 6% mandarin peel powder and observed improvement in fiber, ash, fat, and other phytochemical concentrations. Their experimental results were adequate to draw the conclusion that citrus processing by-products, specifically citrus seeds, have enough potential to enhance the nutritional quality of foods if included at levels that can effectively regulate the technological, physical, and sensory characteristics of developed products. The similar findings from the experimental studies of Rani et al. [26] were also in positive relation with the current one.

Orange seeds can be a crucial supply of cooking oil because they are not susceptible to microbial deterioration or germination. Orange seed's potential for use in both human and animal feeds appears promising. Sweet orange seeds have a reasonably high crude fiber content, which points to the possibility of using them as a viable dietary fiber source. Ash content in sweet orange seed kernels is high; the main minerals are potassium, calcium, sodium, and iron [7]. Recent experiments of Imeneo et al. [54] also provided similar findings as moisture contents of lemon pomace powder-incorporated biscuits were decreased. Citrus seed powder cookies were subjected to sensory analysis by Yilmaz and Karaman [25], which revealed that while appearance scores were comparable to the control sample, taste/flavor attribute scores were poor. The values for fracturability, hardness, and water activity hold true during storage at room temperature for 90 days. Due to their high fiber and flavonoid contents, the orange seed fiber crackers may offer some health benefits to users. Current findings were also in line with the results of Omima et al. [55], as they reported an increase in fiber, protein, and ash contents of strawberry and soy flour-incorporated biscuits.

Wheat flour has recently become more crucial for incorporating fruit and vegetable peels and their powders into customized functional and medicinal food products. Recent research on biscuits made with pumpkin peel powder by Hussain et al. [29] revealed that the ash, fat, and fiber contents of the added peel powder biscuits were higher than those made with plain wheat flour. Additionally, it was claimed that functionalized biscuits made with fruit peels have enough levels of phenolic, flavonoid, carotenoid, and mineral contents. Using various replacement levels of citrus pomace powder, Lagana et al. [27] measured the moisture contents and water activity of biscuits and discovered that these values dramatically increased as the level of citrus pomace powder increased. Due to fiber's ability to retain moisture, researchers connected this rise to the biscuits' higher fiber content.

### 3.5. Mineral Composition of Orange Seed Powder-Incorporated Biscuits

Mean values of important minerals present in control and orange seed powder-incorporated biscuits have been shown in Table 5, from where it can be seen that substituting wheat flour with orange seed powder caused a significant rise in mineral contents of formulated biscuits. Contents of calcium, magnesium, potassium, zinc, manganese, zinc, and selenium in control biscuits were found 20.51 ± 0.08, 17.29 ± 0.04, 46.12 ± 0.05, 1.06 ± 0.01, 1.97 ± 0.01, 0.12 ± 0.01, and 0.11 ± 0.01 mg/100 g, respectively, and replacement of wheat flour with 10% orange seed powder increased values of these minerals to 103.90 ± 0.35, 44.35 ± 0.50, 71.29 ± 0.32, 2.59 ± 0.4, 2.75 ± 0.02, 1.31 ± 0.01, and 2.02 ± 0.05 mg/100 g, respectively. This significant increase in mineral contents of incorporated biscuits was attributed to the high mineral profile of orange seeds.

Minerals are essential for human nutrition that regulate various metabolic processes in the body and also provide support to the skeleton. Higher consumption of cereal-based bakery items has led to the deficiencies of important minerals in humans, which can only be encountered by incorporation of nonwheat flours from plants, fruits, and vegetables into wheat flour for the development of nutritional food products [56]. The high mineral contents in fruits are responsible for the rise in calcium, iron, zinc, and other minerals in incorporated biscuits. Infants and young children need calcium for healthy growth and development, while iron and zinc are essential for metabolism and immunity. Magnesium is another mineral that is abundant in carrots. Magnesium is necessary for the synthesis of bone, protein, new cells, the activation of B vitamins, the relaxation of muscles and nerves, blood coagulation, and many other metabolic processes in the human body [57]. Humans who experience persistent vomiting, malabsorption, or severe diarrhea may become deficient in magnesium. Selenium plays a crucial role in how tocopherols are metabolized and is helpful in the management of protein and calorie deficiencies. A modest zinc shortage is characterized by hypogeusia (reduced perception of taste), poor development, and poor appetite. Iron has a role in the synthesis, packaging, absorption, and breakdown of neurotransmitters into other iron-containing proteins, all of which can directly or indirectly affect brain function. Slip tendon disease results from a deficiency [56, 58]. In other animals, congenital abnormalities in the development of the embryonic bone are brought on by manganese shortage [59]. Phosphorus is necessary for optimal physiological function and is a component of the high-energy storage molecules ADP and ATP. Lack of phosphorus can cause rickets in children and osteomalacia in adults. The integrity of cell structure is preserved by calcium [58, 59].

Sweet orange seeds can be used in salt-restricted diets due to their low sodium content. Nutritionists have always been interested in the iron content of food. Despite its low concentration, iron contents of orange seeds could prove a useful element for consumers. Calcium in accessible form present in orange seeds would provide customers with a decent source of organic calcium [8]. Incorporation of orange seed powders in different food products have been resulted in an increment of mineral contents of food products [7]. Present study results were also in accordance with the previously reported results of Omima et al. [55], regarding the increase in mineral contents of soy and strawberry powder-incorporated biscuits, which proved helpful in meeting the daily demand for children's minerals.

### 3.6. Vitamin Composition of Orange Seed Powder-Incorporated Biscuits

Vitamin composition of control biscuits and orange seed powder-incorporated biscuits have been offered in Table 6. Vitamins that are soluble in fat were found significantly increased as a result of increasing orange seed powder level in biscuits. All B group vitamins, especially B1 and B2, were also found significantly increased in formulated biscuits, and a nonsignificant decrease was found for B1 and B2. Vitamins K and B12, which were not detected in control biscuits, were also observed in orange seed powder-supplemented biscuits.

Fractions like husk, peels, pomaces, leaves, seeds, and stems of fruits and vegetables are typically thrown despite the fact that they may contain nutrients such as carotenoids, dietary fibers, vitamins, minerals, and polyphenols. Utilizing fruits, vegetables, and crop waste to create functional and pharma foods in different sectors is a result of the food industry's growing interest in bioactives [3]. Citrus by-products have been found loaded with bioactives, which include B complex, C, and fat-soluble vitamins, and the incorporation of these citrus by-products in the form of extracts, powders, and as a whole in different food products have provided nutritional foods [60]. An earlier experiment by Cabuk et al. [61] has provided vitamin-enriched egg products supplemented by citrus waste. Citrus waste, which included peels, leaves, and seeds, has been reported to possess ascorbic acid, citric acid, and other important vitamins [62]. Antioxidant-related properties of citrus fruit-enriched food products have been found linked with the presence of phytochemicals present in citrus components like phenolics, minerals, and vitamins [63].

Vitamin E has anti-inflammatory properties in addition to its function as a free radical scavenger, especially in high dosages. The substantial adverse relationship between plasma vitamin E and CVD is supported by growing research. Functional foods rich in vitamin E play a positive role in improving health [64]. Globally, and particularly in developing nations, vitamin C insufficiency is a serious health problem, with scurvy being the result of a severe deficit. Researchers in natural products and chemistry have given fruits a lot of attention as preventive strategies for extremely prevalent noncommunicable diseases because of the numerous pharmacological actions linked with them. It is generally known that the phytochemicals found in fruits and other plant components have synergistic pharmacological actions to strengthen the immune-related systems, which are directly linked to a lower chance of contracting communicable diseases [65].

Traditional biscuits with high fats and sugars, which most consumers do not identify with a healthy diet, can be adjusted, and according to Boobier et al. [66], by reducing salt and sugar while increasing fibers, and essential vitamins, this modification technique turned a conventional food into an innovative and health-promoting one. Youssef [67] successfully increased the vitamin contents of wheat flour biscuits by the addition of wheat germ oil. In a similar study, Makpoul and Ibrahem [52] replaced wheat flour with quinoa seed flour and detected a significant increase in B group vitamins and E, strengthening the current findings. Similar reports were also observed in the experiments of Omima et al. [55], during the development of soy and strawberry powder-incorporated biscuits, which were found high in vitamins.

### 3.7. Sensory Evaluation of Orange Seed Powder-Incorporated Biscuits

Results containing scores of sensory evaluation of different treatment biscuits have been shown in Table 7, from where it can be easily analyzed that for colour and flavor, a 5% replacement level of orange seed powder was preferred by judges. Similarly, a 5% level of orange seed powder got the best scores for texture, taste, and overall acceptability. On the other hand, although higher replacement levels improved the nutritional values of the developed biscuits, their sensory scores significantly decreased for all parameters as overall acceptability scores were just 6.81 ± 0.23 for these biscuits, which were significantly lesser than 7.74 ± 0.16, the scores of 5% orange seed powder-incorporated biscuits. Orange leftovers and the impact of different processing patterns on the bioactive components in different fruit by-product flours were assessed in a recent study. These were found rich in phenolic and flavonoid components, and they also have a strong reducing and antioxidant effect, which could be the key reason behind their use in different food products [68].

Supportive findings were also present in the findings of Hussain et al. [29], witnessing the use of fruits and vegetable powders at lower levels that proved to develop acceptable bakery products. The current study's findings are supported by the sensory analysis of a study by Bolarinwa et al. [69], which found that the bread made with 5% moringa was not significantly different from the bread made with 100% wheat flour in terms of the majority of the quality attributes assessed in their study. Similar findings were also present in the research work by Boriy et al. [70] during the development of pan breads. Another study by Omima et al. [55] reported 10% strawberry powder as the optimum level of replacement for wheat flour biscuits.

Emojorho and Akubor [10] compared the sensory scores of flour blends containing different proportions of orange seed powder and wheat flour and reported similar results. Findings of a recent study by Hussain et al. [28] also witnessed that suitable replacement levels of flour from lemon pomace provided optimum quality biscuits with high nutritional components. Instrumental texture research by Talens et al. [20] revealed that compared to biscuits made with commercial orange fiber, hot air and microwaved dried orange seed fiber produced biscuits with reduced hardness. In order to produce less refined, more nutrient-dense, and more sustainable components for the fiber-enrichment of bakery goods, fiber from citrus seeds can be used to upcycle orange by-products.

Abdel-Naeem et al. [71] found that adding powders made from lemon, orange, grapefruit, and banana peels improved the colour, aroma, look, and tenderness of chicken patties. The judges were pleased with the generated end products, which has opened up new possibilities for the creation of a wide range of dairy, desserts, beverage, confectionary, and meat products using nonwheat powders from medicinal plants. By including citrus pomace powder at various replacement levels, functional biscuits were developed by Lagana et al. [27], and they discovered that adding citrus pomace powder at a 10% replacement level resulted in high-quality, nutrient-dense biscuits with enhanced antioxidant activity. Results from Rani et al. [26] also exposed a trend that was consistent with the findings of the current study. Ojha and Thapa [24] found that biscuits produced with 6% peel powder of mandarin outperformed the control group.

Similar results were found in studies done by Hussain et al. [5] on biscuits made with fruit and vegetable peel powder at a 5% replacement level with optimum acceptance. The presence of more flavoring compounds and polyphenols in orange seed powder may account for the decreased flavor and taste scores of biscuits with 7.5 and 10% orange seed powder replacement, while the presence of more fiber may also contribute to the decreased texture scores with higher orange seed powder levels. Low scores of overall acceptability and appearance are a result of the 10% orange seed powder biscuits' more yellow to greenish hue, which is a result of natural pigments found in the orange seed. After performing chemical and functional tests on these powders, Jurasova and Kukurova [23] created biscuits by substituting 5%, 10%, and 15% of white flour with powders made from lemon and orange by-products and reported 10% use of these powders acceptable. In comparison to lemon waste powder-incorporated biscuits, orange waste powder-incorporated biscuits received substantially higher grades.

Biscuits with orange seed powder in them had almost comparable sensory profiles, with the exception of the flavor's strength, which was stronger for orange seeds and less soft. These biscuits had a little to some degree of orange flavor, little to some degree of sweetness, bitterness, natural flavor, adequate colour, little to some degree of orange fragrance, and adequate crispiness. In keeping with customers' worries about food allergies and related toxicities, the negative impacts of unreal food colours, and the desire to consume foods that are good for their health, biscuit with orange seed powder displayed a typical orange colour [20, 72]. The latter findings concur with those of the present investigation. All of these customer problems could be addressed with orange seed powder.

## 4. Conclusions

The significance of conducting research for its application has been noted as an outcome of the rising global fruit and vegetable processing, food production, and waste generation. As a result, there is increasing interest in fruit and vegetable seeds that contain bioactive chemicals, such as those that are obtained from fruit seeds. Orange seeds were found to be a rich source of fat, ash, fiber, and protein contents when compared with wheat flour, and due to this high proximate composition, incorporation of orange seed powder resulted in a significant increase of these parameters in developed biscuits. Similarly, the presence of important and necessary vitamins in orange seed powder was observed during this study, and comparing amounts of these vitamins with those present in wheat flour revealed that vitamin deficiencies of refined wheat flour and developed products can be overcome by the addition of orange seed powder; as in the current study, a significant rise in vitamins of biscuits was calculated. Sensory evaluation of the developed biscuits revealed that 5% replacement of orange seed powder provided good quality biscuits with acceptable sensorial scores from panellists. In order to provide comprehensive information to determine the proper utilization patterns of these seeds and their incorporation as ingredients in the production of different food items, food supplements, pharma foods, pharmaceuticals, and cosmetics, it is imperative to conduct further analysis of the nutraceutical components and their uses. However, this research can be a useful resource for future research initiatives because further study is required in more extensive areas of fruit seed potentials through the implementation of novel techniques.

## 5. Recommendations

The treatment of waste streams generated by the consumption of fresh and processed fruits has always been a major challenge for food processors. Because the fruit contains physiologically active components that can be isolated and used in the creation of functional food formulations, recent research has shown that no portion of the fruit can be regarded as waste. While the production of juice is the primary goal of the citrus processing businesses, enormous amounts of trash, including seeds, peels, and pomace, pose environmental and health problems if not properly disposed of. Valorization of citrus fruit waste might be a crucial step towards sustainable pharma food development and the environment. It is needed to implement novel and innovative processes and techniques to handle, process, preserve, and utilize these wastes in safe manners, in order to get maximum sustainability with minimum resources. The seeds of exotic tropical orange fruits show their vast potential as a natural supply of food formulation components and additives to enhance the value of contemporary food stuffs.

## Figures and Tables

**Table 1:** Proximate analyses of wheat flour and orange seed powder.

Treatments	Proximate composition (%)				
	Moisture	Ash	Protein	Fat	Fiber
Wheat flour	12.78 ± 0.06^a^	1.21 ± 0.03^b^	11.02 ± 0.12^b^	0.75 ± 0.01^b^	0.87 ± 0.02^b^
Orange seed powder	4.58 ± 0.02^b^	32.58 ± 0.03^a^	16.29 ± 0.32^a^	19.82 ± 0.40^a^	14.95 ± 0.51^a^

Mean scores within the columns followed by the same alphabetical letter are not significantly different (*p* < 0.05).

**Table 2:** Mineral composition of wheat flour and orange seed powder.

Treatments	Mineral composition (mg/100 g)						
	Calcium	Magnesium	Potassium	Zinc	Iron	Manganese	Selenium
Wheat flour	24.56 ± 0.06^b^	112.82 ± 0.10^b^	150.70 ± 0.12^b^	0.32 ± 0.04^b^	0.88 ± 0.03^b^	0.76 ± 0.03^b^	0.62 ± 0.02^b^
Orange seed powder	620.90 ± 2.25^a^	279.45 ± 0.15^a^	234.90 ± 0.50^a^	3.88 ± 0.12^a^	2.12 ± 0.05^a^	2.25 ± 0.04^a^	5.32 ± 0.03^a^

Mean scores within the columns followed by the same alphabetical letter are not significantly different (*p* < 0.05).

**Table 3:** Vitamin analysis of wheat flour and orange seed powder.

Treatments	Vitamins (mg/100 g)									
	A	D	E	K	B1	B2	B3	B6	B9	B12
Wheat flour	3.01 ± 0.02^b^	2.01 ± 0.02^b^	ND	ND	0.14 ± 0.01^a^	4.32 ± 0.06^a^	0.42 ± 0.02^a^	1.68 ± 0.01^a^	2.38 ± 0.03^a^	ND
Orange seed powder	528.25 ± 0.20^a^	24.10 ± 0.05^a^	32.62 ± 0.40^a^	1.68 ± 0.10^a^	0.10 ± 0.01^b^	3.76 ± 0.04^b^	3.10 ± 0.04^a^	11.77 ± 0.04^a^	14.60 ± 0.10^a^	1.42 ± 0.02^a^

Mean scores within the columns followed by the same alphabetical letter are not significantly different (*p* < 0.05). ND: not detected.

**Table 4:** Proximate composition of orange seed powder-incorporated biscuits.

Treatments	Proximate composition (%) of orange seed powder-incorporated biscuits				
	Moisture	Ash	Protein	Fat	Fiber
0% (control)	5.66 ± 0.02^a^	1.63 ± 0.03^e^	7.12 ± 0.07^e^	28.12 ± 0.12^e^	0.38 ± 0.02^e^
2.5% orange seed powder	5.30 ± 0.03^b^	4.45 ± 0.03^d^	7.93 ± 0.10^d^	31.48 ± 0.50^d^	1.91 ± 0.02^d^
5% orange seed powder	5.08 ± 0.01^c^	6.78 ± 0.01^c^	8.76 ± 0.20^c^	33.71 ± 0.40^c^	3.08 ± 0.04^c^
7.5% orange seed powder	4.74 ± 0.04^d^	8.18 ± 0.05^b^	9.59 ± 0.30^b^	35.84 ± 0.62^b^	5.48 ± 0.10^b^
10% orange seed powder	4.48 ± 0.03^e^	9.68 ± 0.04^a^	10.42 ± 0.25^a^	36.90 ± 0.55^a^	6.79 ± 0.12^a^

Mean scores within the columns followed by the same alphabetical letter are not significantly different (*p* < 0.05).

**Table 5:** Mineral composition of orange seed powder-incorporated biscuits.

Treatments	Mineral composition (mg/100 g) of wheat flour and orange seed powder						
	Calcium	Magnesium	Potassium	Zinc	Iron	Manganese	Selenium
0% (control)	20.51 ± 0.08^e^	17.29 ± 0.04*e*	46.12 ± 0.05^e^	1.06 ± 0.01^e^	1.97 ± 0.01^e^	0.12 ± 0.01^e^	0.11 ± 0.01^e^
2.5% orange seed powder	35.52 ± 0.50^d^	25.42 ± 0.20^d^	52.28 ± 0.10^d^	1.41 ± 0.03^d^	2.18 ± 0.03^d^	0.56 ± 0.02^d^	0.72 ± 0.02^d^
5% orange seed powder	57.48 ± 0.80^c^	32.29 ± 0.34^c^	58.71 ± 0.40^c^	1.79 ± 0.04^c^	2.37 ± 0.02^c^	0.89 ± 0.2^c^	1.05 ± 0.04^c^
7.5% orange seed powder	86.51 ± 0.15^b^	37.10 ± 0.25^b^	64.50 ± 0.36^b^	2.16 ± 0.02^b^	2.56 ± 0.03^b^	1.08 ± 0.03^b^	1.63 ± 0.03^b^
10% orange seed powder	103.90 ± 0.35^a^	44.35 ± 0.50^a^	71.29 ± 0.32^a^	2.59 ± 0.4^a^	2.75 ± 0.02^a^	1.31 ± 0.01^a^	2.02 ± 0.05^a^

Mean scores within the columns followed by the same alphabetical letter are not significantly different (*p* < 0.05).

**Table 6:** Vitamin composition of orange seed powder-incorporated biscuits.

Treatments	Vitamins (mg/100 g)									
	A	D	E	K	B1	B2	B3	B6	B9	B12
0% (control)	1.37 ± 0.03^e^	1.41 ± 0.04^e^	0.48 ± 0.03^e^	ND	0.31 ± 0.02^a^	1.01 ± 0.04^a^	0.94 ± 0.03^e^	0.24 ± 0.01^e^	1.23 ± 0.03^e^	ND
2.5% orange seed powder	12.56 ± 0.55^d^	3.05 ± 0.05^d^	0.74 ± 0.03^d^	0.03 ± 0.01^d^	0.30 ± 0.02^a^	0.98 ± 0.03^a^	1.39 ± 0.04^d^	0.56 ± 0.02^d^	1.58 ± 0.02^d^	0.03 ± 0.01^d^
5% orange seed powder	19.36 ± 0.40^c^	4.61 ± 0.03^c^	0.98 ± 0.04^c^	0.16 ± 0.02^c^	0.29 ± 0.01^a^	0.83 ± 0.02^b^	1.64 ± 0.06^c^	0.69 ± 0.02^c^	1.93 ± 0.05^c^	0.14 ± 0.01^c^
7.5% orange seed powder	25.78 ± 0.20^b^	5.76 ± 0.06^b^	1.39 ± 0.03^b^	0.35 ± 0.03^b^	0.27 ± 0.03^a^	0.80 ± 0.01^b^	1.88 ± 0.02^b^	0.83 ± 0.01^b^	2.48 ± 0.06^b^	0.23 ± 0.01^b^
10% orange seed powder	32.05 ± 0.25^a^	7.12 ± 0.03^a^	1.81 ± 0.04^a^	0.52 ± 0.04^a^	0.27 ± 0.03^a^	0.69 ± 0.03^c^	2.07 ± 0.03^a^	0.96 ± 0.02^a^	2.79 ± 0.02^a^	0.39 ± 0.03^a^

Mean scores within the columns followed by the same alphabetical letter are not significantly different (*p* < 0.05). ND: not detected.

**Table 7:** Sensory evaluation of orange seed powder-incorporated biscuits.

Substitution plan of biscuits	Sensory evaluation parameters of orange seed powder-incorporated biscuits				
	Colour	Flavor	Taste	Texture	Overall acceptability
0% (control)	7.51 ± 0.15^b^	7.27 ± 0.38^c^	7.40 ± 0.21^c^	7.65 ± 0.12^b^	7.29 ± 0.18^b^
2.5% orange seed powder	7.60 ± 0.36^b^	7.65 ± 0.22^b^	7.92 ± 0.71^b^	7.87 ± 0.15^a^	7.68 ± 0.22^a^
5% orange seed powder	7.92 ± 0.17^a^	8.25 ± 0.43^a^	8.13 ± 0.12^a^	7.93 ± 0.13^a^	7.74 ± 0.16^a^
7.5% orange seed powder	7.12 ± 0.14^c^	7.20 ± 0.33^c^	7.80 ± 0.32^b^	7.12 ± 0.18^c^	7.36 ± 0.18^b^
10% orange seed powder	6.20 ± 0.16*d*	6.12 ± 0.13^d^	6.68 ± 0.41^d^	6.55 ± 0.09^d^	6.81 ± 0.23^c^

Mean scores within the columns followed by the same alphabetical letter are not significantly different (*p* < 0.05).

## Data Availability

Upon request from any individual or organization, the corresponding authors will give data pertinent to this work.

## Additional Points

*Practical Applications*. While the production of juice is the primary goal of the citrus processing businesses, enormous amounts of trash, including seeds, peels, and pomace, pose environmental and health problems if not properly disposed of. Valorization of citrus fruit waste might be a crucial step towards sustainable pharma food development and the environment. The seeds of orange fruits show their vast potential as an expected supply of components and additives to add value to contemporary foods. Potential applications in the food, pharmaceutical, and cosmetic industries include the nutritional and bioactive components.
